# Precision neuro-oncology: a pilot analysis of personalized treatment in recurrent glioma

**DOI:** 10.1007/s00432-022-04050-w

**Published:** 2022-08-12

**Authors:** Lazaros Lazaridis, Teresa Schmidt, Christoph Oster, Tobias Blau, Daniela Pierscianek, Jens T. Siveke, Sebastian Bauer, Hans-Ulrich Schildhaus, Ulrich Sure, Kathy Keyvani, Christoph Kleinschnitz, Martin Stuschke, Ken Herrmann, Cornelius Deuschl, Björn Scheffler, Sied Kebir, Martin Glas

**Affiliations:** 1 Department of Neurology and Center for Translational Neuro- and Behavioral Sciences (C-TNBS), Division of Clinical Neurooncology, University Medicine Essen, University Duisburg-Essen, Essen, Germany; 2 German Cancer Consortium (DKTK), Partner Site University Medicine Essen, Hufelandstr. 55, 45147 Essen, Germany; 3 DKFZ-Division Translational Neurooncology at the West German Cancer Center (WTZ), DKTK Partner Site, University Medicine Essen, University Duisburg-Essen, Essen, Germany; 4 German Cancer Consortium (DKTK), German Cancer Research Center (DKFZ), Heidelberg, Germany; 5 Institute of Neuropathology, University Medicine Essen, University Duisburg-Essen, Essen, Germany; 6 Department of Neurosurgery and Spine Surgery, University Medicine Essen, University Duisburg-Essen, Essen, Germany; 7 Bridge Institute of Experimental Tumor Therapy, West German Cancer Center (WTZ), University Medicine Essen, University Duisburg-Essen, Essen, Germany; 8 Division of Solid Tumor Translational Oncology, German Cancer Consortium (DKTK, Partner Site Essen) and German Cancer Research Center, DKFZ, Heidelberg, Germany; 9 Department of Medical Oncology, West German Cancer Center (WTZ), University Medicine Essen, University Duisburg-Essen, Essen, Germany; 10 Department of Medical Oncology, Sarcoma Center, University Medicine Essen, University Duisburg-Essen, Essen, Germany; 11 Institute of Pathology, University Medicine Essen, University Duisburg-Essen, Essen, Germany; 12 Department of Radiotherapy, University Medicine Essen, University Duisburg-Essen, Essen, Germany; 13 Department of Nuclear Medicine, University Medicine Essen, University Duisburg-Essen, Essen, Germany; 14 Institute for Diagnostic and Interventional Radiology and Neuroradiology, University Medicine Essen, University Duisburg-Essen, Essen, Germany

**Keywords:** Precision oncology, Targeted therapy, Molecularly matched targeted therapy, Neuro-oncology, Glioma

## Abstract

**Purpose:**

When brain cancer relapses, treatment options are scarce. The use of molecularly matched targeted therapies may provide a feasible and efficacious way to treat individual patients based on the molecular tumor profile. Since little information is available on this strategy in neuro-oncology, we retrospectively analyzed the clinical course of 41 patients who underwent advanced molecular testing at disease relapse.

**Methods:**

We performed Sanger sequencing, targeted next generation sequencing, and immunohistochemistry for analysis of potential targets, including programmed death ligand 1, cyclin D1, phosphorylated mechanistic target of rapamycin, telomerase reverse transcriptase promoter mutation, cyclin-dependent kinase inhibitor 2A/B deletion, or *BRAF-V600E* mutation. In selected patients, whole exome sequencing was conducted.

**Results:**

The investigation included 41 patients, of whom 32 had isocitrate dehydrogenase (*IDH*) wildtype glioblastoma. Molecular analysis revealed actionable targets in 31 of 41 tested patients and 18 patients were treated accordingly (matched therapy group). Twenty-three patients received molecularly unmatched empiric treatment (unmatched therapy group). In both groups, 16 patients were diagnosed with recurrent *IDH* wildtype glioblastoma. The number of severe adverse events was comparable between the therapy groups. Regarding the *IDH* wildtype glioblastoma patients, median progression-free survival (mPFS) and median overall survival (mOS) were longer in the matched therapy group (mPFS: 3.8 versus 2.0 months, *p* = 0.0057; mOS: 13.0 versus 4.3 months, *p* = 0.0357).

**Conclusion:**

These encouraging data provide a rationale for molecularly matched targeted therapy in glioma patients. For further validation, future study designs need to additionally consider the prevalence and persistence of actionable molecular alterations in patient tissue.

**Supplementary Information:**

The online version contains supplementary material available at 10.1007/s00432-022-04050-w.

## Introduction

Novel systemic treatment options are urgently needed in the field of neuro-oncology. For glioblastoma patients, clinical outcome remains poor and effective systemic treatment options beyond temozolomide are scarce. Especially at disease recurrence, the standards of care are not well defined (Weller and Le Rhun 2020).

In the 2016 World Health Organization (WHO) Classification of Tumors of the Central Nervous System, a set of molecular markers has been implemented in clinical routine (Louis et al. 2016). With the current fifth edition of the WHO classification, published in 2021, the impact of molecular markers with relevance for diagnosis and treatment for primary brain tumors further increased (Louis et al. 2021). To address intra- and interindividual tumor heterogeneity—a known phenomenon of malignant intracranial tumors (Körber et al. 2019)—personalized molecularly matched therapy strategies are the next logical step in anticancer treatment.

The identification of actionable molecular drivers is increasingly gaining traction for multiple different cancer entities and has become a central part in cancer treatment (Bedard et al. 2020). Next generation sequencing (NGS) techniques, among others (e.g. Sanger sequencing, immunohistochemistry, fluorescence in situ hybridization—FISH) led to a better understanding of brain tumors at the molecular level and resulted in the identification of promising actionable targets—such as *BRAF-V600E* mutations—revealing novel opportunities for targeted treatment. Despite the progress made over the past years, the therapeutic relevance of both driver mutations and also the increasing number of variants of unknown significance in a cancer-specific context are in their infancy, particularly for brain cancer (Carr et al. 2016). Nevertheless, tumor-agnostic investigations postulate a superior efficacy of a molecularly matched targeted therapy over molecularly unmatched treatments. In a retrospective cancer-agnostic investigation with approximately 1500 patients, Tsimberidou et al. reported higher rates of response and longer overall survival rates for patients with molecularly matched targeted therapy compared to patients who received molecularly unmatched empiric therapy (Tsimberidou et al. 2017). On the other hand, there is an ongoing debate on how far the cancer-agnostic precision oncology approach can be taken, as the cellular context remains important for the vast majority of genomic variants (Photopoulos 2020). In recent years, interdisciplinary tumor boards have begun to implement molecularly based treatment suggestions in the field of neuro-oncology, particularly for the notoriously treatment-resistant relapse of disease. There is a paucity of data regarding the outcome of these treatment suggestions.

In prior clinical glioblastoma trials, targeted treatments failed to improve overall survival in predominantly molecularly unselected patient cohorts (Weller and Le Rhun 2020). However, the known glioblastoma-inherent inter- and intraindividual tumor heterogeneity requires a strong patient selection for the application of personalized molecularly matched targeted therapies. The ongoing N2M2/NOA-20 trial (EudraCT number: 2015-002,752-27) will provide important data about the efficacy of molecularly matched targeted therapies in first-line glioblastoma treatment (Wick et al. 2019).

In our retrospective analysis of recurrent glioma patients, we were curious to determine pilot data on the feasibility of molecularly based treatment suggestions.

## Methods

### Study design

All patients with recurrent disease under oncologic treatment at the Division of Clinical Neurooncology, Department of Neurology at the University Hospital Essen, who underwent advanced molecular testing from January 2017 until December 2020 were considered eligible for this retrospective analysis. The following inclusion criteria had to be met:

1. Adult patients diagnosed with recurrent glioma.
2. Availability of information on the pathohistological brain tumor diagnosis as per the WHO Classification of Tumors of the Central Nervous System from 2016 (Louis et al. 2016).
3. Performed advanced molecular testing (see below) at the Institute of Neuropathology of the University Hospital Essen or by an external provider of genetic diagnostics and sequencing services.

Decisions to perform advanced molecular analysis for patients with recurrent glioma were made by treating physicians. Data were discussed in the molecular tumor board of the University Hospital Essen and treatment recommendations were based on the criteria for molecularly matched targeted therapy (see below). As most targeted treatments do not have a label for brain cancer, approval for reimbursement by health insurances was requested for each individual patient, which requires an additional medical review by insurances. Before starting therapy, we extensively informed patients about their individual baseline situation, the side effect profile of the corresponding drug, and the available treatment alternatives in detailed individual visits. In a few patients, Karnofsky Performance Score (KPS) was 50% and almost exclusively driven by motor deficits (such as hemiparesis or nonfluent aphasia), whereas the cognitive function was well preserved allowing for the patients to provide reliable informed consent. These patients were considered to receive a further line of treatment, if an estimated life expectancy of at least six months was to be assumed. Treatment response was determined according to the updated response assessment criteria for high-grade gliomas (Wen et al. 2010). Toxicity was assessed according to the Common Terminology Criteria for Adverse Events (CTCAE, Version 5). Magnetic resonance imaging (MRI) scans to ascertain treatment response were performed every 8–12 weeks.

This study was approved by the local ethics committee of the University Duisburg-Essen (application number: 20-9431-BO).

### Advanced molecular analysis

We performed immunohistochemistry on programmed death ligand 1 (PD-L1), phosphorylation of mechanistic target of rapamycin (p-mTOR), and cyclin D1, Sanger sequencing for telomerase reverse transcriptase (*TERT*) promoter, FISH for cyclin-dependent kinase inhibitor 2A/B (*CDKN2A/B*) deletion, and panel-based NGS including key genetic alterations associated with tumor proliferation such as *BRAF-V600E* mutation. Furthermore, whole exome sequencing (WES) using Illumina’s NovaSeq 6000 system with a read length of 2 × 100 base pairs and an output of 12 GB per sample with matched WES from blood (germ-line control) were conducted in 18 patients.

### Molecularly matched targeted therapy

Molecular alterations were considered actionable, if clinical or compelling preclinical evidence of a predictive benefit from a specific therapy (in any cancer type) had been reported in the past (Pishvaian et al. 2020). Actionability was assessed considering published evidence according to Leichsenring et al. (Leichsenring et al. 2019) as well as the evidence for blood–brain barrier penetration of the molecularly matched targeted drug and the availability in Germany. Also, the patient’s medical history (including standard therapies, off-label therapies, and enrollment into specific clinical trials) was considered. In case of multiple molecularly matched targeted treatment options, a shortlist of ranked therapy options was generated on a case-by-case basis. If no actionable target was detected or molecularly matched targeted treatment could not be performed for other reasons (see below for detailed information), a molecularly unmatched empiric treatment decision was implemented.

### Statistics

We used the Kaplan–Meier estimator to assess the survival function from lifetime data. Concerning the evaluation of the progression-free survival (PFS) and overall survival (OS), the time period of interest extended from the time point of latest MRI-defined recurrence before onset of investigated therapy until the next MRI indicating repeat recurrence. Before the initiation of treatment, we ruled out putative pseudoprogression by subsequent MRI and/or positron emission tomography (PET) imaging according to the updated response assessment criteria for high-grade gliomas (Wen et al. 2010). If progression or death had not occurred at the time of analysis (August 31, 2021), the patient was considered censored for survival analysis. For data visualization, GraphPad Prism version 9.2.0 (GraphPad Software Inc., San Diego, USA) and Affinity Designer version 1.9.0 (Serif Europe, West Bridgford, UK) were used.

## Results

### Patient characteristics

In total, 41 patients received advanced molecular analysis, of whom 18 (44%) received a molecularly matched targeted treatment recommendation (henceforth defined as the matched therapy group) and 23 (56%) were treated according to a molecularly unmatched empiric treatment decision (henceforth defined as the unmatched therapy group). In both groups, 16 patients were diagnosed with recurrent isocitrate dehydrogenase (*IDH*) wildtype glioblastoma. All patient characteristics are listed in Tables 1, 2. Detailed clinical and molecular information for every single patient from the matched therapy group and the unmatched therapy group is listed in Supplementary Table S1 and Supplementary Table S2. The therapy groups were balanced for age and KPS. In both groups, the investigated treatment was administered between the first and fifth disease recurrence with median treatment onset at second recurrence. In both therapy groups, 61% of the patients had no additional treatment (surgery, radiotherapy, tumor-treating fields) besides systemic medical therapy during the therapy line of interest. Concerning the *IDH* wildtype glioblastoma patients, 56% in the matched therapy group versus 63% in the unmatched therapy group received no additional treatment during the therapy line of interest.

**Table 1:** Patient characteristics from all patients of the matched therapy group and the unmatched therapy group

	Matched therapy group	Unmatched therapy group
*n*	18	23
Age, range (median)	21–74 (55)	22–68 (54)
Gender, *n*		
Male	14 (78%)	11 (48%)
Female	4 (22%)	12 (52%)
KPS at therapy onset, range (median)	50–90% (70%)	50–90% (70%)
Histopathological diagnosis, *n*		
Glioblastoma WHO IV	16 (89%)	18 (78%)
Anapl. Astrocytoma WHO III	2 (11%)	3 (13%)
Diff. Astrocytoma WHO II	–	2 (9%)
Treatment line, *n*		
1. Recurrence	1 (6%)	1 (4%)
2. Recurrence	11 (61%)	14 (61%)
3. Recurrence	3 (16%)	6 (27%)
4. Recurrence	2 (11%)	1 (4%)
5. Recurrence	1 (6%)	1 (4%)
Investigated tissue, *n*		
Therapy line of investigated therapy	2 (11%)	3 (13%)
One therapy line prior to investigated therapy	10 (55%)	8 (35%)
Two therapy lines prior to investigated therapy	5 (28%)	10 (44%)
Three therapy lines prior to investigated therapy	–	–
Four therapy lines prior to investigated therapy	1 (6%)	1 (4%)
Five therapy lines prior to investigated therapy	–	1 (4%)
Additional treatment in investigated therapy line, *n*		
Surgery	6 (33%)	4 (17%)
Radiotherapy	1 (5%)	4 (17%)
TTFields	3 (17%)	3 (13%)
None	11 (61%)	14 (61%)
Duration of treatment, weeks (median)	4–48 (8)	4–22 (8)
Time interval between recurrence MRI and onset of investigated therapy, weeks (median)	1–5 (3)	1–5 (2)

*Anapl* anaplastic, *Diff* diffuse, *KPS* Karnofsky Performance Score, *MRI* magnetic resonance imaging, *TTFields* Tumor Treating Tields, *WHO* World Health Organization.

**Table 2:** Patient characteristics for all isocitrate dehydrogenase (*IDH*) wildtype glioblastoma patients of the matched therapy group and the unmatched therapy group

	Matched therapy group	Unmatched therapy group
*n*	16	16
Age, range (median)	21–72 (55)	38–68 (56)
Gender, *n*		
Male	13 (81%)	7 (44%)
Female	3 (19%)	9 (56%)
KPS at therapy onset, range (median)	50–90% (70%)	50–90% (70%)
*MGMT* promoter status, *n*		
Methylated	2 (13%)	7 (44%)
Unmethylated	14 (87%)	9 (56%)
Treatment line, *n*		
1. Recurrence	1 (6%)	1 (6%)
2. Recurrence	11 (69%)	11 (69%)
3. Recurrence	3 (19%)	4 (25%)
4. Recurrence	1 (6%)	–
Investigated tissue, *n*		
Therapy line of investigated therapy	2 (12%)	3 (19%)
One therapy line prior to investigated therapy	8 (50%)	5 (31%)
Two therapy lines prior to investigated therapy	4 (25%)	8 (50%)
Three therapy lines prior to investigated therapy	1 (6%)	–
Four therapy lines prior to investigated therapy	1 (6%)	–
Additional treatment in investigated therapy line, *n*		
Surgery	6 (38%)	3 (19%)
Radiotherapy	1 (6%)	2 (12%)
TTFields	3 (19%)	3 (19%)
None	9 (56%)	10 (63%)
Duration of treatment, weeks (median)	4–40 (8)	4–16 (8)
Time interval between recurrence MRI and onset of investigated therapy, weeks (median)	1–5 (3)	1–5 (2)

*KPS* Karnofsky Performance Score, *MGMT* O(6)-methylguanine-DNA methyltransferase, *MRI* magnetic resonance imaging, *TTFields* Tumor Treating Fields

**Fig. 1:**
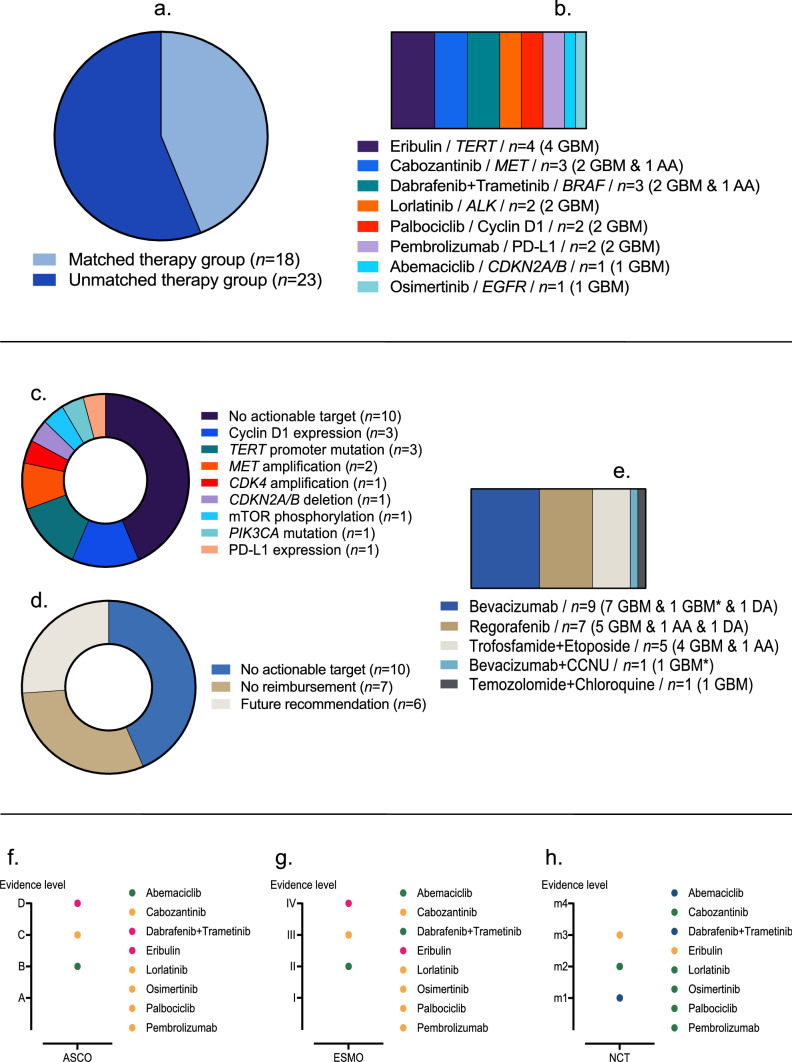
Basal characteristics of the molecularly advanced analyzed patient cohort. Eighteen (44%) patients received molecularly matched targeted therapy (matched therapy group) and in 23 (56%) patients a molecularly unmatched empiric treatment decision (unmatched therapy group) was implemented (**a**). Overall, a total of eight different molecularly matched targeted therapy schemes were administered (**b**). In the unmatched therapy group ten (43%) patients had no actionable target detected (**c**), which was—among others—the major cause for not receiving a molecularly matched targeted therapy. For seven patients no reimbursement from the corresponding health insurance could be obtained and in six patients under empiric treatment, molecularly matched targeted treatment was a future recommendation at the time of data cutoff (**d**). In the unmatched therapy group, five different therapy schemes were administered (**e**). Treatment decisions in the matched therapy group were mostly made on the basis of evidence from other tumor entities. The corresponding evidence levels according to available classifications from the American Society of Clinical Oncology (ASCO), the European Society for Medical Oncology (ESMO), and the National Center for Tumor Diseases Heidelberg (NCT) are shown in (**f**–**h**). *AA* anaplastic astrocytoma, *ALK* anaplastic lymphoma kinase, *ASCO* American Society of Clinical Oncology, *CDK4* cyclin-dependent kinase 4, *CDKN2A/*B cyclin-dependent kinase inhibitor 2A/B, *DA* diffuse astrocytoma, *EGFR* epidermal growth factor receptor, *ESMO* European Society for Medical Oncology, *GBM* isocitrate dehydrogenase (*IDH*) wildtype glioblastoma, GBM* *IDH* mutant glioblastoma, *NCT* National Center for Tumor Diseases Heidelberg, *mTOR* mechanistic target of rapamycin, *PIK3CA* phosphatidylinositol 3-kinase catalytic subunit alpha, *PD-L1* programmed death ligand 1, *TERT *telomerase reverse transcriptase

Thirty-one (76%) patients had an actionable molecular target and 18 (44%) were treated with a molecularly matched targeted therapy, whereas 23 (56%) were treated according to a molecularly unmatched empiric treatment decision instead (Fig. 1a). Fifteen from 18 (83%) patients in the matched therapy group had more than one actionable target. In total, eight different molecularly matched targeted treatment schemes were administered (Fig. 1b). Eribulin, a fully synthetic macrocyclic ketone analogue of the marine natural product halichondrin B and a specific inhibitor of *TERT*-RNA-dependent RNA polymerase, was used in patients with *TERT* promoter mutation according to published preclinical data (Takahashi et al. 2019). Cabozantinib, a small molecule multi-tyrosine kinase inhibitor against vascular endothelial growth factor receptor (*VEGFR*) and *MET* was used in patients with *MET* amplification, as it was shown to be effective in the treatment of lung cancer (D’Arcangelo et al. 2019). The combination of dabrafenib plus trametinib represents standard of care for *BRAF-V600E*-mutant melanoma and lung cancer patients (Long et al. 2015; Planchard et al. 2017) and was applied in case of *BRAF-V600E* mutation on the basis of several case reports in brain tumors (Johanns et al. 2018). Lorlatinib, an orally administered inhibitor of anaplastic lymphoma kinase (*ALK*) and *ROS1*, was administered in patients with *ALK* rearrangement pursuant to the findings observed in lung cancer (Shaw et al. 2020). Palbociclib, a cyclin-dependent kinase 4/6 (*CDK4/6*) inhibitor, was administered in patients with homozygous *CDKNA2/B* deletion due to published evidence from its use in metastatic breast cancer (Finn et al. 2016). Pembrolizumab was used in patients with PD-L1 expression due to published evidence from the treatment of glioblastoma (Reardon et al. 2021). Abemaciclib was used on the basis of homozygous *CDKN2A/B* deletion according to the results of the interim analysis of the Individualized Screening Trial of Innovative Glioblastoma Therapy (INSIGhT) (Wen et al. 2020). Osimertinib, a highly potent small molecule inhibitor of epidermal growth factor receptor (*EGFR*), was used in the presence of an *EGFR* mutation as had been used previously in the successful treatment of lung cancer (Soria et al. 2018). In the unmatched therapy group, no actionable target could be detected in ten (44%) patients (Fig. 1c). The reasons for the other 13 patients in the unmatched therapy group with detected actionable molecular targets for not receiving a molecularly matched targeted therapy were lack of reimbursement approval from the health insurance in seven patients, and for six patients currently under active molecularly unmatched empiric treatment, reimbursement for a molecularly matched targeted treatment option was approved by the corresponding health insurance, making it a future treatment recommendation in case of repeat tumor progression (Fig. 1d). In the unmatched therapy group, five different treatment schemes were administered (Fig. 1e). Figure 1f–h shows a synoptical display of the corresponding evidence levels according to Leichsenring et al. (Leichsenring et al. 2019) regarding the use of the molecularly matched targeted therapies. As indicated, most of the applied molecularly matched targeted drugs were based on level C (according to the American Society of Clinical Oncology—ASCO—classification), level III (according to the European Society for Medical Oncology—ESMO—classification) or level m2 (according to the National Center for Tumor Diseases Heidelberg—NCT—classification) evidence (Fig. 1f-h).

There were three patients in the matched therapy group with treatment durations of at least 24 weeks (six months). Two of these patients received treatment with the combination of dabrafenib plus trametinib and one patient received treatment with cabozantinib. The patient treated with cabozantinib had the longest duration of treatment in the matched therapy group (48 weeks), he was diagnosed with an *IDH* mutant glioblastoma and received cabozantinib treatment at second disease recurrence. The patients with the second and third longest duration of treatment in the matched therapy group (40 and 24 weeks) were diagnosed with *IDH* wildtype glioblastomas and received treatment with the combination of dabrafenib plus trametinib at fourth disease recurrence (patient with the second longest treatment duration) and at first disease recurrence (patient with the third longest treatment duration).

### Treatment response

**Fig. 2:**
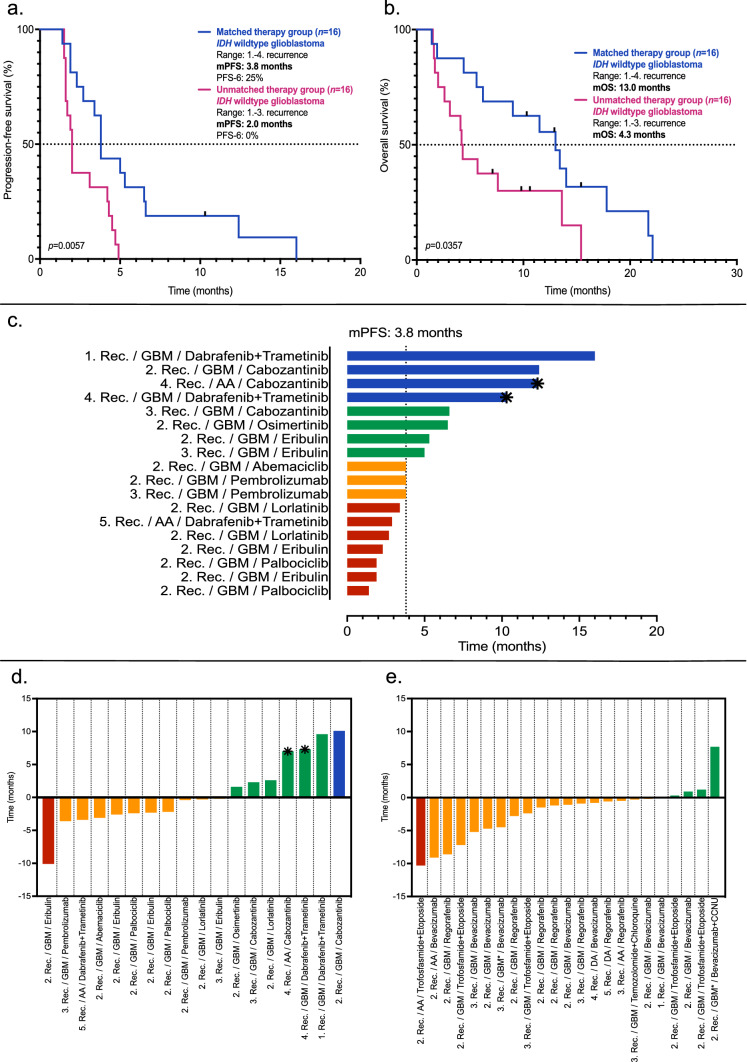
Kaplan–Meier curves for the isocitrate dehydrogenase (*IDH*) wildtype glioblastoma patients of the matched and unmatched therapy group and individual outcomes. Median progression-free survival and median overall survival were longer in the matched therapy group compared with the unmatched therapy group for *IDH* wildtype glioblastoma patients (**a**, **b**). Individual progression-free survival for each patient from the matched therapy group is illustrated by the swimmer’s plot in (**c**). For every patient in the matched therapy group (**d**) and in the unmatched therapy group (**e**), the progression-free survival difference between the therapy line of molecularly matched targeted therapy (matched therapy group) or molecularly unmatched empiric treatment (unmatched therapy group) and the prior therapy line is depicted. Each column represents the difference of one single patient of the matched therapy group (**d**) or the unmatched therapy group (**e**). A positive value means that the investigated treatment resulted in a prolonged progression-free survival compared to the previously administered treatment. A negative value means that the investigated treatment resulted in a shortened progression-free survival compared to the previously administered treatment. *Data censored at cutoff; *AA* anaplastic astrocytoma, *DA* diffuse astrocytoma, *GBM *isocitrate dehydrogenase (*IDH*) wildtype glioblastoma, GBM* *IDH* mutant glioblastoma, *mOS* median overall survival, *mPFS* median progression-free survival, *Rec* recurrence

The median progression-free survival (mPFS) for the *IDH* wildtype glioblastoma patients was 3.8 months in the matched therapy group and 2.0 months in the unmatched therapy group (HR: 2.39, 95% CI: 1.1–5.1, *p* = 0.0057, Fig. 2a). PFS-6 was 25% (matched therapy group) versus 0% (unmatched therapy group). Median overall survival (mOS) for the *IDH* wildtype glioblastoma patients was 13.0 months in the matched therapy group versus 4.3 months in the unmatched therapy group (HR: 2.14, 95% CI: 0.94–4.88, *p* = 0.0357, Fig. 2b).

As shown in Fig. 2c, individual PFS analysis in the matched therapy group revealed longest PFS intervals for treatment with the combination of dabrafenib plus trametinib and for treatment with cabozantinib. The longest PFS (16 months) was observed upon treatment with dabrafenib plus trametinib in an *IDH* wildtype glioblastoma patient at first recurrence. The poorest outcome was noted for patients treated with palbociclib. Two of three patients who received a combination of dabrafenib plus trametinib and all patients who received a single-drug treatment with cabozantinib or osimertinib had longer PFS durations than mPFS (3.8 months) in the matched therapy group.

Individual PFS differences between the treatment line in which the molecularly matched targeted therapy was administered and the treatment line immediately before revealed a longer PFS under molecularly matched targeted therapy in seven patients with the largest extension in an *IDH* wildtype glioblastoma patient after treatment with cabozantinib at second disease recurrence (+ 10.1 months). All cabozantinib-treated patients and two of three patients treated with the combination of dabrafenib plus trametinib had a longer PFS under molecularly matched targeted therapy. The most prominent PFS shortening could be observed for a patient after treatment with eribulin (-10.1 months). The individual PFS differences for the matched therapy group are shown in Fig. 2d. Regarding the unmatched therapy group, in only five patients a PFS extension was present. The individual PFS differences for the unmatched therapy group are shown in Fig. 2e.

### Toxicity

Treatment-related death occurred neither in the matched therapy group nor in the unmatched therapy group. The number of adverse events of CTCAE grade III or higher was comparable in both groups. Table 3 synoptically displays the toxicity data of all patients subdivided into matched and unmatched therapy group.

**Table 3:** Toxicity observed for all patients according to the Common Terminology Criteria for Adverse Events (CTCAE, Version 5)

	Matched therapy group	Unmatched therapy group
*n*	18	23
Treatment-related deaths, *n*	–	–
Total events CTCAE ≥ III, *n*	23	25
Hematotoxicity CTCAE ≥ III, *n*	11 (48%)	9 (39%)
Neutropenia	5 (45%)	2 (22%)
Thrombopenia	-	2 (22%)
Lymphopenia	5 (45%)	5 (56%)
Pancytopenia	1 (10%)	-
Nonhematotoxicity CTCAE ≥ III, *n*	12 (52%)	16 (61%)
GGT elevation	3 (25%)	2 (13%)
GPT elevation	–	2 (13%)
Thromboembolic event	1 (8%)	–
Seizure	1 (8%)	1 (6%)
Hand–foot syndrome	1 (8%)	2 (13%)
Elevation of INR	1 (8%)	–
Wound complication	1 (8%)	1 (6%)
Infection	4 (35%)	3 (19%)
Hypertension	–	1 (6%)
Lipase elevation	–	1 (6%)
Proteinuria	–	1 (6%)
Hyperbilirubinemia	–	1 (6%)
Hypernatremia	–	1 (6%)

*GGT* gamma-glutamyltransferase, *GPT* glutamate pyruvate transaminase, *INR* international normalized ratio

## Discussion

Our pilot data indicate that molecularly matched targeted brain tumor treatment is well tolerated and associated with prolonged disease control and longer survival compared to patients who received molecularly unmatched empiric therapy or patients with tumors lacking those molecular markers and genomic alterations that were selected for this study. Our retrospective analysis considered in an unbiased way every local glioma patient at disease recurrence whose tumor tissue received advanced molecular diagnostics from January 2017 until December 2020. We observed actionable molecular alterations in 76% of the cases, of whom 44% received molecularly matched targeted treatment. For other solid cancer entities, actionable targets were detected in 26% (pancreatic cancer), 47% (extrahepatic cholangiosarcoma), and 40% (in a tumor-agnostic study by Tourneau et al.) of investigated patients (Pishvaian et al. 2020; Lowery et al. 2018; Le Tourneau et al. 2015). In comparison, the high number of actionable targets in our cohort of glioma patients may implicate a great variety of different molecularly matched targeted treatment options in the field of neuro-oncology. However, it must be taken into account that in some previous trials only molecular alterations within distinct molecular pathways were considered, whereas in our analysis we considered any putatively actionable molecular alteration with report of clinical or compelling preclinical evidence of a predictive benefit from a specific therapy (in any cancer type).

Previous reports on molecularly matched targeted treatment in the field of neuro-oncology had already focused on feasibility, but these studies were conducted in small patient cohorts and/or did not consider toxicity or systematic comparison to a valid control group (Byron et al. 2018; Blumenthal et al. 2016; Kessler et al. 2020). Notably, some of the retrospectively included patients even underwent molecular testing for first-line treatment of glioblastoma (Kessler et al. 2020). Our study revolved around defining treatment options for recurrent glioblastoma. Considering the natural course of disease, the need for rational treatment options is highest at the time of tumor relapse, but there is no standard-of-care treatment available (Weller et al. 2021, 2013; Chaul-Barbosa and Marques 2019). To address this dilemma, every here reported patient received personalized treatment according to advanced molecular analysis and based on molecular tumor board consensus decision. Subsequently, survival times increased by a factor of two to three when molecularly matched targeted treatment was employed. The benefit occurred relative to molecularly unmatched empiric treatment that the respective control group of patients received. Molecularly unmatched empiric treatment in the glioblastoma subcohort resulted in a mPFS of 2.0 months and mOS of 4.3 months, which compares to the survival noted in the lomustine control arm of the REGOMA trial (Lombardi et al. 2019). Notably, the survival times (mPFS and mOS) of glioblastoma patients in our molecularly matched targeted therapy group exceeded any of the reported control arm data considerably, even though most of their clinical courses were more advanced (Batchelor et al. 2013; Wick et al. 2017). Thus, we noted an encouraging therapy response to molecularly matched targeted treatment.

Considering the high diagnostic effort, it must nevertheless be questioned why the effect of molecularly matched targeted treatment has not turned out to be higher. Apparently, the dynamic nature of glioblastoma, including cell and genotype heterogeneity, microenvironmental interactions, subclonal dynamics and plasticity remain a tough competitor in the management of recurrent disease (Qazi et al. 2017; Reinartz et al. 2017; Körber et al. 2019; Barthel et al. 2019; Bi et al. 2020; Schäfer et al. 2019). Furthermore, the blood–brain barrier may impact the efficacy and therapeutic window for the drugs that were used. For the molecularly matched targeted drugs used in this analysis with known ability to effectively pass the blood–brain barrier (e.g. cabozantinib, lorlatinib, abemaciclib, and osimertinib), published evidence indicates safety and clinical benefit in intracranial anticancer activity against brain metastases originating from different cancer entities (Peverelli et al. 2019; Bauer et al. 2020; Tolaney et al. 2020; Park et al. 2020). In this context, it is important to mention that for osimertinib and cabozantinib, the potential of blood–brain barrier penetrance has been investigated exclusively in previous preclinical studies (Colclough et al. 2021; Abdelaziz and Vaishampayan 2017), whereas for abemaciclib and lorlatinib previous human studies have been performed in which the drugs were detected in sufficient concentration in the cerebrospinal fluid (Patnaik et al. 2016; Shaw et al. 2017). We would view molecularly matched targeted therapy as one additional component of modern precision medicine that needs to incorporate clinically robust and relevant predictive biomarkers, innovative therapy monitoring including liquid and frequent tissue biopsies as well as combinatorial and sequential treatment strategies, patient-derived experimental disease models and co-clinical trials, and artificial intelligence-guided predictive computational models (Rajewsky et al. 2020; Aldape et al. 2019). It remains to be questioned whether the effect of molecularly matched targeted treatment can be improved by combining multiple drugs.

The findings of a study by Körber et al.—inclucing analysis of genomes, transcriptomes, and methylomes in paired primary (untreated) and recurrent (following initial treatment) tumor tissue samples from 50 patients with *IDH* wildtype glioblastoma—imply that standard therapy exerted little selective pressure on (most) recurrent tumors (Körber et al. 2019). Körber et al. postulated that the vast majority of driver mutations were acquired prior to initial diagnosis. These results are in tandem with the results of the study by Barthel et al. In this study, temporally separated DNA sequencing data and matched clinical annotation from 222 glioma patients were analyzed. Based on this analysis, little evidence of recurrence-specific gene alterations was found (Barthel et al. 2019). These findings contrast with the description by Kim et al. of divergent glioblastoma recurrences that share few genetic alterations with the primary tumor (Kim et al. 2015). Johnson et al. reported for low-grade gliomas—based on paired exome sequencing of 23 patients—that in 43% of cases at least half of the mutations in the initial tumor were undetected at recurrence (Johnson et al. 2014). Similar results were shown also by the work of Schäfer et al.: Based on a paired tissue analysis of 34 glioblastoma patients, a clinically relevant longitudinal heterogeneity of molecular target expression was detected leading to the assumption that patient tissue as recent as possible should be used for advanced molecular analysis (Schäfer et al. 2019). However, it is evident that even the most sensitive assay delivers only a snapshot at a distinct time point in the evolution of cancer. Clearly, new models of glioblastoma should address both prevalence and persistence of actionable molecular alterations in patient tissue and should broaden analysis beyond a single treatment-naïve sample at diagnosis to capture the evolution of recurrent, treatment-resistant disease (Qazi et al. 2017). In this study, we attempted to ensure that the molecular testing results were obtained from the recurrent tumor tissue and that treatment according to those alterations was conducted in the same line of treatment. In most patients, however, this approach failed, leaving a great deal of uncertainty as to whether the target was present at the time of treatment. We also need to consider that the patients in our pilot study were heavily pretreated; the majority of patients had received a molecularly matched targeted therapy at higher lines of treatment up to the fifth tumor recurrence. Nonetheless, we observed some particularly well-performing drugs: abemaciclib, a potent *CDK4/6* inhibitor with good brain penetration approved for breast cancer, and the combination of dabrafenib/trametinib, a *BRAF*/mitogen-activated protein kinase kinase (*MEK*) inhibitor approved for the adjuvant treatment of melanoma with *BRAF-V600E* or *-V600K* mutations. There are early indications from other reports that these two drugs act favorably in glioblastoma patients (Wen et al. 2020; Johanns et al. 2018).

Lastly, limitations inherent to the performed analysis should be mentioned. The retrospective study design, small sample size and heterogeneous cohort, inconsistent disease stages, lack of randomization, and diverse types of administered drugs cannot allow simplified conclusions on efficacy. Furthermore, it has to be questioned if the presence of a distinct actionable molecular target is linked per se with a favorable prognosis in brain cancer. For future studies, a putative selection bias has to be ruled out by a matched-pair control cohort. Furthermore, it remains unknown whether a specific target represents the real tumor driver in a specific tumor entity.

However, we have observed a thorough effect of molecularly matched targeted therapy. The data from our pilot study provide a very reasonable rationale for follow-up of a larger cohort of molecularly stratified glioblastoma patients in a prospective controlled trial.

## Supplementary Information

Below is the link to the electronic supplementary material.

Supplementary file1 (DOCX 27 kb)

Supplementary file2 (DOCX 29 kb)

## Data Availability

The datasets generated during and/or analyzed during the current study are available from the corresponding author on reasonable request.
