# Supplementary material for: Precision neuro-oncology: a pilot analysis of personalized treatment in recurrent glioma
Source: J Cancer Res Clin Oncol. 2022 Aug 12;149(7):3513–26. doi: 10.1007/s00432-022-04050-w (PMC10314879; doi:10.1007/s00432-022-04050-w)
Supplement: Supplementary file 1 — Supplementary file1 (DOCX 27 kb) [file 432_2022_4050_MOESM1_ESM.docx]

# Precision Neuro-oncology - A Pilot Analysis of Personalized Treatment in Recurrent Glioma

Journal of Cancer Research and Clinical Oncology

Lazaros Lazaridis, Teresa Schmidt, Christoph Oster, Tobias Blau, Daniela Pierscianek, Jens T. Siveke, Sebastian Bauer, Hans-Ulrich Schildhaus, Ulrich Sure, Kathy Keyvani, Christoph Kleinschnitz, Martin Stuschke, Ken Herrmann, Cornelius Deuschl, Björn Scheffler*, Sied Kebir*, Martin Glas*

**Correspondence:**

Prof. Dr. Martin Glas, Department of Neurology, Division of Clinical Neurooncology, University Medicine Essen, University Duisburg-Essen, Hufelandstr. 55, Essen, 45147, Germany. Phone 0049 201 723 6520, Fax 0049 201 723 6985, E-mail: [Martin.Glas@uk-essen.de](mailto:Martin.Glas@uk-essen.de)

**Shared last authorship*

| **Age [years]** | **Gender** | **KPS at therapy onset [%]** | **Histopathological diagnosis** | **Treatment line** | **Investigated tissue** | **Molecular target** | **Molecularly matched targeted therapy** | **Additional treatment in investigated therapy line** |
| --- | --- | --- | --- | --- | --- | --- | --- | --- |
| 21 | Female | 90 | Glioblastoma WHO IV | 1. Recurrence | -1 | *BRAF* | Dabrafenib+Trametinib | Surgery+Radiotherapy |
| 28 | Male | 80 | Glioblastoma WHO IV | 4. Recurrence | -4 | *BRAF* | Dabrafenib+Trametinib | None |
| 35 | Male | 90 | Glioblastoma WHO IV | 2. Recurrence | 0 | *MET* | Cabozantinib | Surgery+TTFields |
| 40 | Male | 80 | Glioblastoma WHO IV | 2. Recurrence | -1 | *CDKN2A/B* | Abemaciclib | Surgery |
| 43 | Male | 70 | Anapl. Astrocytoma WHO III | 4. Recurrence | -2 | *MET* | Cabozantinib | None |
| 43 | Female | 60 | Glioblastoma WHO IV | 2. Recurrence | 0 | *EGFR* | Osimertinib | Surgery+TTFields |
| 46 | Male | 50 | Glioblastoma WHO IV | 3. Recurrence | -2 | *MET* | Cabozantinib | None |
| 47 | Male | 90 | Glioblastoma WHO IV | 2. Recurrence | -2 | *ALK* | Lorlatinib | None |
| 55 | Male | 70 | Glioblastoma WHO IV | 2. Recurrence | -1 | *TERT* | Eribulin | None |
| 55 | Male | 60 | Glioblastoma WHO IV | 2. Recurrence | -1 | *ALK* | Lorlatinib | TTFields |
| 56 | Male | 90 | Glioblastoma WHO IV | 3. Recurrence | -1 | PD-L1 | Pembrolizumab | Surgery |
| 64 | Female | 90 | Glioblastoma WHO IV | 2. Recurrence | -2 | PD-L1 | Pembrolizumab | None |
| 67 | Male | 70 | Glioblastoma WHO IV | 3. Recurrence | -1 | *TERT* | Eribulin | None |
| 69 | Male | 60 | Glioblastoma WHO IV | 2. Recurrence | -2 | Cyclin D1 | Palbociclib | None |
| 70 | Male | 70 | Glioblastoma WHO IV | 2. Recurrence | -1 | *TERT* | Eribulin | None |
| 70 | Male | 80 | Glioblastoma WHO IV | 2. Recurrence | -1 | *TERT* | Eribulin | Surgery |
| 72 | Male | 60 | Glioblastoma WHO IV | 2. Recurrence | -1 | Cyclin D1 | Palbociclib | None |
| 74 | Female | 70 | Anapl. Astrocytoma WHO III | 5. Recurrence | -1 | *BRAF* | Dabrafenib+Trametinib | None |

**Supplementary Table S1.** Detailed clinical and molecular information for every single patient from the matched therapy group.

0: Therapy line of investigated therapy; -1: One therapy line prior to investigated therapy; -2: Two therapy lines prior to investigated therapy; -3: Three therapy lines prior to investigated therapy; -4: Four therapy lines prior to investigated therapy; *ALK*: Anaplastic lymphoma kinase; Anapl.: Anaplastic; *CDKN2A/B*: Cyclin-dependent kinase inhibitor 2A/B; *EGFR*: Epidermal growth factor receptor; KPS: Karnofsky Performance Score; PD-L1: Programmed death ligand 1; *TERT*: Telomerase reverse transcriptase; TTFields: Tumor Treating Fields; WHO: World Health Organization
