# Supplementary material for: Precision neuro-oncology: a pilot analysis of personalized treatment in recurrent glioma
Source: J Cancer Res Clin Oncol. 2022 Aug 12;149(7):3513–26. doi: 10.1007/s00432-022-04050-w (PMC10314879; doi:10.1007/s00432-022-04050-w)
Supplement: Supplementary file 2 — Supplementary file2 (DOCX 29 kb) [file 432_2022_4050_MOESM2_ESM.docx]

# Precision Neuro-oncology - A Pilot Analysis of Personalized Treatment in Recurrent Glioma

Journal of Cancer Research and Clinical Oncology

Lazaros Lazaridis, Teresa Schmidt, Christoph Oster, Tobias Blau, Daniela Pierscianek, Jens T. Siveke, Sebastian Bauer, Hans-Ulrich Schildhaus, Ulrich Sure, Kathy Keyvani, Christoph Kleinschnitz, Martin Stuschke, Ken Herrmann, Cornelius Deuschl, Björn Scheffler*, Sied Kebir*, Martin Glas*

**Correspondence:**

Prof. Dr. Martin Glas, Department of Neurology, Division of Clinical Neurooncology, University Medicine Essen, University Duisburg-Essen, Hufelandstr. 55, Essen, 45147, Germany. Phone 0049 201 723 6520, Fax 0049 201 723 6985, E-mail: [Martin.Glas@uk-essen.de](mailto:Martin.Glas@uk-essen.de)

**Shared last authorship*

| **Age [years]** | **Gender** | **KPS at therapy onset [%]** | **Histopathological diagnosis** | **Treatment line** | **Investigated tissue** | **Molecular target** | **Reason for not applying molecularly matched targeted therapy** | **Molecularly unmatched empiric therapy** | **Additional treatment in investigated therapy line** |
| --- | --- | --- | --- | --- | --- | --- | --- | --- | --- |
| 22 | Male | 80 | Anapl. Astrocytoma WHO III | 2. Recurrence | -1 | Cyclin D1 expression | No reimbursement | Trofosfamide+Etoposide | Radiotherapy |
| 38 | Female | 90 | Glioblastoma WHO IV | 2. Recurrence | -1 | No target detected | No target detected | Regorafenib | TTFields |
| 43 | Female | 90 | Diff. Astrocytoma | 5. Recurrence | -5 | No target detected | No target detected | Regorafenib | None |
| 45 | Male | 60 | Diff. Astrocytoma | 4. Recurrence | -4 | No target detected | No target detected | Bevacizumab | None |
| 45 | Female | 60 | Glioblastoma WHO IV | 2. Recurrence | -2 | No target detected | No target detected | Bevacizumab | None |
| 45 | Male | 80 | Glioblastoma WHO IV | 2. Recurrence | -1 | No target detected | No target detected | Trofosfamide+Etoposide | Surgery+Radiotherapy |
| 45 | Male | 80 | Glioblastoma WHO IV | 2. Recurrence | -2 | Cyclin D1 expression | No reimbursement | Bevacizumab+CCNU | Surgery |
| 47 | Male | 90 | Glioblastoma WHO IV | 3. Recurrence | -2 | *TERT* promoter mutation | Future recommendation | Regorafenib | None |
| 50 | Female | 90 | Anapl. Astrocytoma WHO III | 2. Recurrence | -1 | Cyclin D1 expression | No reimbursement | Bevacizumab | None |
| 52 | Female | 50 | Glioblastoma WHO IV | 3. Recurrence | -2 | No target detected | No target detected | Bevacizumab | None |
| 53 | Female | 70 | Glioblastoma WHO IV | 3. Recurrence | -1 | No target detected | No target detected | Bevacizumab | Radiotherapy |
| 54 | Male | 70 | Glioblastoma WHO IV | 2. Recurrence | -1 | *PIK3CA* mutation | Future recommendation | Trofosfamide+Etoposide | None |
| 55 | Male | 80 | Glioblastoma WHO IV | 2. Recurrence | -2 | *MET* amplification | Future recommendation | Bevacizumab | TTFields |
| 55 | Male | 50 | Glioblastoma WHO IV | 3. Recurrence | -2 | No target detected | No target detected | Temozolomide+Chloroquine | None |
| 55 | Male | 70 | Glioblastoma WHO IV | 2. Recurrence | -1 | *CDK4* amplification | No reimbursement | Regorafenib | Surgery+Radiotherapy |
| 57 | Female | 50 | Glioblastoma WHO IV | 2. Recurrence | -1 | *TERT* promoter mutation | Future recommendation | Bevacizumab | None |
| 57 | Female | 80 | Anapl. Astrocytoma WHO III | 3. Recurrence | -2 | *MET* amplification | No reimbursement | Regorafenib | None |
| 59 | Male | 70 | Glioblastoma WHO IV | 3. Recurrence | 0 | No target detected | No target detected | Trofosfamide+Etoposide | None |
| 59 | Male | 70 | Glioblastoma WHO IV | 2. Recurrence | -2 | mTOR phosphorylation | Future recommendation | Bevacizumab | None |
| 60 | Female | 70 | Glioblastoma WHO IV | 1. Recurrence | 0 | *CDKN2A/B* deletion | Future recommendation | Bevacizumab | Surgery |
| 64 | Female | 90 | Glioblastoma WHO IV | 2. Recurrence | -2 | *TERT* promoter mutation | No reimbursement | Regorafenib | None |
| 66 | Female | 60 | Glioblastoma WHO IV | 2. Recurrence | -2 | PD-L1 expression | No reimbursement | Regorafenib | TTFields |
| 68 | Female | 60 | Glioblastoma WHO IV | 2. Recurrence | 0 | No target detected | No target detected | Trofosfamide+Etoposide | None |

**Supplementary Table S2.** Detailed clinical and molecular information for every single patient from the unmatched therapy group.

0: Therapy line of investigated therapy; -1: One therapy line prior to investigated therapy; -2: Two therapy lines prior to investigated therapy; -3: Three therapy lines prior to investigated therapy; -4: Four therapy lines prior to investigated therapy; -5: Five therapy lines prior to investigated therapy; Anapl.: Anaplastic; *CDK4*: Cyclin-dependent kinase 4; *CDKN2A/B*: Cyclin-dependent kinase inhibitor 2A/B; Diff.: Diffuse; KPS: Karnofsky Performance Score; mTOR: Mechanistic Target of Rapamycin; *PIK3CA*: Phosphatidylinositol 3-kinase catalytic subunit alpha*;* PD-L1: Programmed death ligand 1; *TERT*: Telomerase reverse transcriptase; TTFields: Tumor Treating Fields; WHO: World Health Organization
